# Supplementary material for: Rates and Determinants of Hospital-Acquired Infection among ICU Patients Undergoing Cardiac Surgery in Developing Countries: Results from EMERGENCY’NGO’s Hospital in Sudan
Source: Antibiotics (Basel). 2022 Sep 9;11(9):1227. doi: 10.3390/antibiotics11091227 (PMC9494959; doi:10.3390/antibiotics11091227)

SUPPLEMENTARY

RATES AND DETERMINANTS OF HOSPITAL-ACQUIRED INFECTIONS AMONG ICU PATIENTS UNDERGOING CARDIAC SURGERY IN DEVELOPING COUNTRIES: RESULTS FROM THE EMERGENCY'NGO HOSPITAL IN SUDAN

Ornella Spagnolello,[1,2] Silvia Fabris,[3] Gina Portella,[1] Dimiana Raafat Shafig Saber,[1] Elena Giovanella,[1] Manahel Badr Saad,[1] Martin Langer,[1] Massimo Ciccozzi,[3] Gabriella d'Ettorre,[2] Giancarlo Ceccarelli.[2,4]

1. Intensive Care Unit, Salam Centre for Cardio Surgery, EMERGENCY'NGO, Khartoum, Sudan

2. Department of Public Health and Infectious Diseases, University of Rome "*La Sapienza*", Rome, Italy

3. Medical Statistics and Epidemiology Unit, Campus Bio-Medico University, Rome, Italy

4. Migrant and Global Health Research Organization – Mi-HeRO, Rome, Italy

DEFINITIONS

Hospital-acquired infections, Blood Stream Infection (BSI), Ventilator Associated Pneumonia (VAP), Urinary Tract Infection (UTI) were defined in accordance with the following guidelines:

- *European Centre for Disease Prevention and Control. European Surveillance of Healthcare-Associated Infections in Intensive Care Units - HAI-Net ICU protocol, version 1.02. Stockolm. ECDC. 2015. Available at https://www.ecdc.europa.eu/en/publications-data/european-surveillance-healthcare-associated-infections-intensive-care-units-hai, last accessed on 10/8/2022*
- *Torres A, Niederman MS, Chastre J, Ewig S, Fernandez-Vandellos P, Hanberger H, Kollef M, Li Bassi G, Luna CM, Martin-Loeches I, Paiva JA, Read RC, Rigau D, Timsit JF, Welte T, Wunderink R. International ERS/ESICM/ESCMID/ALAT guidelines for the management of hospital-acquired pneumonia and ventilator-associated pneumonia: Guidelines for the management of hospital-acquired pneumonia (HAP)/ventilator-associated pneumonia (VAP) of the European Respiratory Society (ERS), European Society of Intensive Care Medicine (ESICM), European Society of Clinical Microbiology and Infectious Diseases (ESCMID) and Asociación Latinoamericana del Tórax (ALAT). Eur Respir J. 2017 Sep 10;50(3):1700582. doi: 10.1183/13993003.00582-2017. PMID: 28890434.*

Table S1. Descriptive statistics among infected and non-infected patients: Wilcoxon-Mann-Whitney p-values, increase in OR and model characteristics.

	Non infected	Infected	p-value	Increase in OR (95% CI)	AIC	pseudoR^2	univariate predictor p-value
Days in ICU - mean (SD)	2.8 (2)	10.4 (9.4)	< 0.001	1.6 (1.5 - 1.8)	348.9	0.35	< 0.001
Days with CVC - mean (SD)	2.81 (2.1)	11.3 (11.2)	< 0.001	1.6 (1.4 - 1.7)	348.8	0.35	< 0.001
Days with foley - mean (SD)	3 (2.4)	10.6 (8.9)	< 0.001	1.5 (1.4 - 1.6)	362.5	0.32	< 0.001
Days with arterial line - mean (SD)	2.8 (2.3)	10.1 (9.1)	< 0.001	1.5 (1.4 - 1.7)	368.1	0.31	< 0.001
Days with adrenalin - mean (SD)	0.4 (0.9)	2.9 (4.5)	< 0.001	1.9 (1.6 - 2.3)	440	0.17	< 0.001
Days with noradrenalin - mean (SD)	0.4 (0.9)	2.5 (3.8)	< 0.001	1.8 (1.5 - 2.1)	458.5	0.14	< 0.001
Days with steroid - mean (SD)	0.05 (0.4)	0.7 (1.8)	< 0.001	1.9 (1.4 - 2.5)	505.4	0.06	< 0.001
Days on CVVH - mean (SD)	0.1 (0.5)	1 (3)	< 0.001	1.7 (1.3 - 2.1)	505.3	0.06	< 0.001
n° foley - n (%)	1 (0.1)	1.2 (0.5)	< 0.001	39.3 (8.8 - 175.2)	483.3	0.1	< 0.001
n° arterial line - n (%)	1 (0.2)	1.6 (0.9)	< 0.001	10.3 (5.9 - 17.9)	447.5	0.16	< 0.001
n° CVC - n (%)	1 (0.2)	1.8 (1.2)	< 0.001	13.8 (7.4 - 25.9)	406.4	0.24	< 0.001
Steroids (yes) - n (%)	11 (2.1)	17 (19.5)	< 0.001	9.9 (4.4 - 21.8)	504.1	0.06	< 0.001
Noradrenalin (yes) - n (%)	136 (25.9)	56 (64.4)	< 0.001	5.1 (3.2 - 8.1)	484.8	0.09	< 0.001
Adrenalin (yes) - n (%)	1 (0.1)	4 (4.6)	< 0.001	22.3 (2.5 - 202.2)	525.1	0.02	0.0057

OT (yes) - n (%)	460 (87.8)	65 (74.7)	0.002	0.4 (0.2 - 0.7)	525.6	0.02	0.001
Reintubation (yes) - n (%)	6 (1.1)	10 (11.5)	< 0.001	11.1 (3.7 - 33.1)	510.4	0.02	< 0.001
Tracheostomy (yes) - n (%)	0	5 (5.7)	< 0.001	0 (0 - 0)	516.7	0.13	0.979

ICU = Intensive Care Unit; CVVH = Continuous Veno-Venous Hemofiltration; CVC = Central Venous Catheter; SD = Standard Deviation; OT = Operating Theatre; OR = Odds Ratio; AIC = Akaike's information criterion

Figure S1. Correlations among significant risk factors, ordered by clusters, all the correlation tests are significant with α < 0.05.

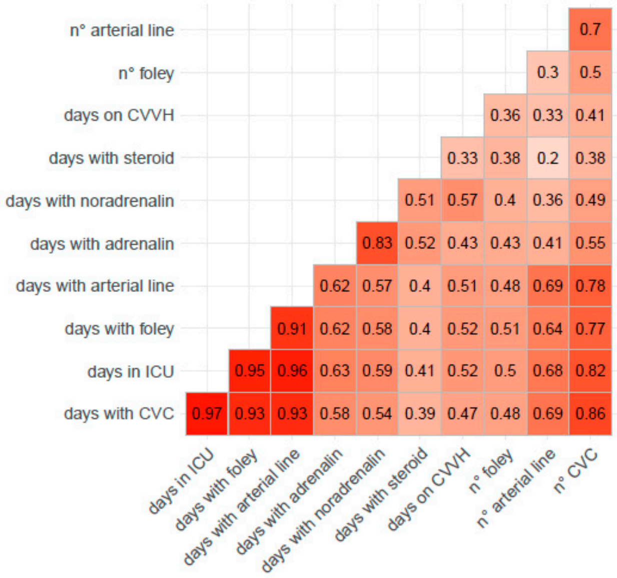

ICU = Intensive Care Unit; CVVH = Continuous Veno-Venous Hemofiltration; CVC = Central Venous Catheter

Figure S2. Multiple logistic regression: ROC curve. The Figure represents ROC curve and the optimal threshold to maximize both accuracy measures, the Area Under the Curve (AUC) is equal to 0.90. Predictive features highlight high model specificity equal to 96.4%.[1] Due to the low prevalence of infection, the model have low sensitivity (SE=36.8%) and NPV higher than PPV (equal to 82.3% and 76.9%, respectively: Mcnemar's Test returned p<0.001.

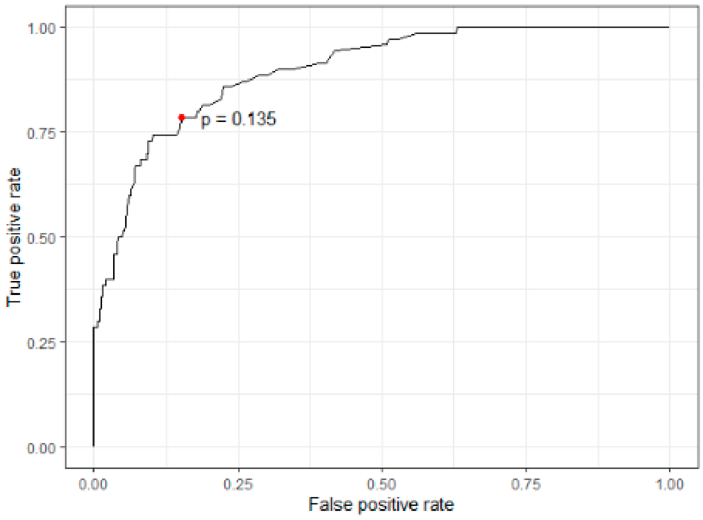

Supplement: Supplementary file 1 [file antibiotics-11-01227-s001.zip › antibiotics-1868887-supplementary.pdf]
